# Morphological and molecular examination of the ciliate family Lagynusidae (Protista, Ciliophora, Prostomatea) with descriptions of two new genera and two new species from China

**DOI:** 10.1007/s42995-023-00174-1

**Published:** 2023-05-29

**Authors:** Limin Jiang, Congcong Wang, Saleh A. Al-Farraj, Hunter N. Hines, Xiaozhong Hu

**Affiliations:** 1 College of Fisheries, and Key Laboratory of Mariculture, Ministry of Education, Ocean University of China, Qingdao, 266003 China; 2 Institute of Evolution and Marine Biodiversity, Ocean University of China, Qingdao, 266003 China; 3 Department of Zoology, College of Science, King Saud University, Riyadh, 11451 Saudi Arabia; 4 Harbor Branch Oceanographic Institute, Florida Atlantic University, Fort Pierce, FL 34982 USA

**Keywords:** Ciliate, Morphology, New genus, New species, Phylogeny, 18S rRNA gene

## Abstract

Ciliates in the class Prostomatea play an important role in the global microbial loop due to their significant abundances and broad feeding strategies at the foundation of food webs. Despite their importance in ecosystems, the taxonomy and systematics of this group of ciliates has long been poorly understood, with this being especially true for members of the family Lagynusidae. Here we examine four lagynusids collected from sandy beaches in China, using silver-staining and 18S rRNA gene sequencing techniques. These investigations revealed two new genera and two new species and provided details for two little known forms: *Penardella marina* gen. nov., sp. nov., *Apolagynus cucumis* (as reported by Penard. Études sur les infusoires d’eau douce. Georg and Cie, Genève, 1922) gen. nov., comb. nov., *Lagynus minutus* sp. nov., and *Lagynus elegans* (Engelmann in Z Wiss Zool 11:347–393, 1862) Quennerstedt (Acta Univ Lund 4:1–48, 1867). *Penardella* gen. nov. can be morphologically distinguished by having more than three dikinetidal perioral kineties. *Apolagynus* gen. nov. differs from the closely related genus *Lagynus* in the absence of a conspicuous neck-like region. The ciliature of *Apolagynus cucumis* is revealed here for the first time, which demonstrates the classification of this species within Lagynusidae. Furthermore, *Apolagynus binucleatus* (Jiang et al., 2021) comb. nov. is established according to the new finding. The results of our phylogenetic analyses based on the 18S rRNA gene support the establishment of two new genera and indicate that Lagynusidae is monophyletic, which further strengthens its valid taxonomic status.

## Introduction

Protistans are integral members of aquatic ecosystems and play a vital role at the foundational level of food webs. Unicellular eukaryotic microorganisms such as microalgae contribute to around half of all photosynthetic activity on earth, and some dinoflagellates and diatoms can cause algal blooms incurring deleterious environmental events (Nagasaki 2008; Worden et al. 2015). Many protists have made important contributions as model organisms in foundational biological research, such as the ciliate *Tetrahymena thermophila* (Ekanem et al. 2004; Xiong et al. 2012). Ciliophora is a monophyletic lineage of protists, and its members exhibit extremely high morphological and genetic diversity (Dragesco 1960; Hu et al. 2019; Kahl 1930; Li et al. 2022; Ma et al. 2022; Song et al. 2009; Wang et al. 2022a, b).

Ciliates in the class Prostomatea Schewiakoff, 1896 often occur in microplankton communities, sometimes with high abundance, and feed on a wide variety of organisms, from bacteria to microalgae (Dragesco 1960; Foissner et al. 1994; Gurdebeke et al. 2018; Hu et al. 2019; Kahl 1930; Lynn 2008; Song et al. 2009; Wang et al. 2022a, b). Due to their relatively simple morphology, prostomateans have fewer morphological characters that can be used for species descriptions when compared with highly specialized ciliates, such as the Spirotrichea (Hu et al. 2019; Song et al. 2009; Wang et al. 2021). The main taxonomically informative characteristics of prostomateans are their cell size and shape, number of macronuclei, brosse position, and presence or absence of extrusomes and caudal cilia (Dragesco 1960; Foissner et al. 1994; Kahl 1930). However, many of these morphological features often overlap between species and thus render identification and characterization of species difficult. Molecular sequences for species within several genera of prostomateans remain sparse and is an area that would benefit from future global sampling efforts.

The first investigations of prostomateans can be traced back to the report of *Coleps hirtus* by Müller (1786). Over the ensuing two centuries, ciliates belonging to the family Colepidae have been the most studied within the class (Chen et al. 2009, 2010, 2016; Dragesco 1960, 1966; Dragesco and Dragesco-Kernéis 1986; Foissner et al. 1994, 2008; Huttenlauch 1987; Pröschold et al. 2021). However, most prostomateans have only cursory original descriptions based exclusively on observations of live cells, with most of these occurring well before the molecular era (Carey 1992; Dragesco 1960; Kahl 1930). Only a handful of species have benefited from descriptions using modern techniques (Foissner 2021; Frantal et al. 2022; Jiang et al. 2021b, 2022; Sonntag et al. 2022). There are also some ultrastructural studies of this group, which revealed details of several important characteristics such as the brosse and the extrusomes (Bardele 1999; Hiller 1991, 1992, 1993; Hiller and Bardele 1988; Lipscomb and Riordan 2012). It is clear that both morphological examinations of the ciliature and molecular sequencing are needed to help elucidate the taxonomy and systematics within ciliates, but such data are often absent, such as for the majority of prostomateans (Gao et al. 2016; Liu et al. 2015; Wright and Colorni 2002; Yi et al. 2010; Zhang et al. 2014). Due to the incompleteness of morphological and morphometric data, as well as the paucity of 18S rRNA sequences, more than half of prostomatean species remain questionable or highly confusing in terms of their circumscription and phylogenetic placement.

Lagynusids are typically elongated, the cell being divided into the “head”, annular neck-like region, and “trunk”, which is an unusual feature in prostomateans (Foissner et al. 1995; Jiang et al. 2021b; Sola et al. 1990). When established, Lagynusidae included only one monotypic genus *Lagynus* Quennerstedt, 1867, with *Lagynus elegans* (Engelmann, 1862) Quennerstedt, 1867 as the type species. Later, two species, *Lagynus cucumis* (Penard, 1922) Foissner, 1987 and *Lagynus binucleatus* Jiang et al., 2021, were added (Foissner 1987; Jiang et al. 2021b; Sola et al. 1990). In terms of 18S rRNA gene sequence data, only that of *L. binucleatus* are available (Jiang et al. 2021b). The lack of further molecular data has hindered the progress of phylogenetic studies of the family Lagynusidae.

In the present study, we provided novel morphological and morphometric data as well as the first 18S rRNA gene sequences of four microaerophilic species of Lagynusidae. We found that some species of lagynusids deviate distinctly from the type species or lack important characteristics (e.g., a conspicuous neck-like region and rod-shaped extrusomes). Thus, we revised the genus *Lagynus* and proposed the establishment of two new genera, *Penardella* and *Apolagynus*, based on complete morphological observations. We described two new species, *P. marina* and *L. minutus*, and made two new combinations, *A. cucumis* and *A. binucleatus*. Phylogenetic analyses based on these new sequences were also carried out to investigate the systematic positions of the four species and to examine the evolutionary relationships among the three genera of the family Lagynusidae, which will shed further light onto the systematics of this group of ciliates.

## Results

### ZooBank registration

This article: urn: lsid: zoobank.org:pub:

A919AE25-F54B-4991-9EA3-FC444964B3FD.

*Penardella* gen. nov.:

urn:lsid:zoobank.org:act:37F1CA03-DF4D-4ED7-923B-BFE662C3D3BF

*Apolagynus* gen nov.:

urn:lsid:zoobank.org:act:EE67F090-22DB-4CD8-8A6B-6D1DBD74909A

*Penardella marina* gen. nov., sp. nov.:

urn:lsid:zoobank.org:act:9CDB80B8-4BA8-4A38-B209-9FDE7B4BC19D.

*Apolagynus cucumis* (Penard, 1922) gen. nov., comb. nov.:

urn:lsid:zoobank.org:act:BB86941B-EA1A-46EA-B34D-34F7D2BD2F0C.

*Lagynus minutus* sp. nov.:

urn:lsid:zoobank.org:act:116520E9-92F7-41A2-8DEC-28D3C7D8065F.

### Taxonomy

Class: Prostomatea Schewiakoff, 1896

Order: Prorodontida Corliss, 1974

Family: Lagynusidae Sola et al., 1990 nom. emend.

### Genus: *Penardella* gen. nov.

**Diagnosis** Generally clavate Lagynusidae; neck-like region slightly contractile, encircled by inconspicuous furrows; more than three dikinetidal perioral kineties.

**Dedication** The genus name is dedicated to Eugène Penard, a celebrated Swiss protozoologist, in acknowledgement of his great contribution to the taxonomy of ciliates. Feminine gender.

**Type species** *Penardella marina* gen. nov., sp. nov.

### ***Penardella marina*** gen. nov., sp. nov. (Figs. 2, 3; Table 1)

**Table 1:** Morphometric data on *Penardella marina* sp. nov., *Apolagynus cucumis* comb. nov., *Lagynus minutus* sp. nov., and the Chinese population of *Lagynus elegans*

Characteristics	Species	Min	Max	M	Mean	SD	CV	*n*
Cell length (μm)	*P. marina*	165	273	236	232.2	28.6	12.3	25
	*A. cucumis*	87	110	103	100.8	6.7	6.7	25
	*L. minutus*	46	65	51	52.5	5.5	10.5	25
	*L. elegans*	88	120	102	101.2	8.9	8.8	25
Cell width (μm)	*P. marina*	25	66	47	47.8	8.5	17.8	25
	*A. cucumis*	20	32	25	25.1	3.3	13.0	25
	*L. minutus*	14	23	17	17.2	2.1	12.0	25
	*L. elegans*	22	42	31	31.0	5.3	17.2	25
Ratio of cell length to cell width	*P. marina*	4	7	5	5.0	0.9	18.1	25
	*A. cucumis*	3	5	44	4.1	0.5	12.6	25
	*L. minutus*	3	3	3	3.0	0.2	6.9	25
	*L. elegans*	3	4	3	3.3	0.4	12.4	25
Macronucleus length (μm)	*P. marina*	77	102	88	87.9	7.5	8.5	17
	*A. cucumis*	14	21	18	18.1	2.0	10.8	25
	*L. minutus*	8	15	11	11.1	1.5	13.6	23
	*L. elegans*	18	33	23	23.5	3.9	16.5	25
Macronucleus width (μm)	*P. marina*	13	24	20	19.1	2.9	15.5	17
	*A. cucumis*	10	17	13	13.0	2.1	15.9	25
	*L. minutus*	4	7	5	5.4	0.8	14.5	23
	*L. elegans*	8	17	11	11.3	1.9	17.0	25
Ratio of macronucleus length to macronucleus width	*P. marina*	4	6	5	5.0	0.5	11.3	17
	*A. cucumis*	1	2	2	1.4	0.3	21.6	25
	*L. minutus*	2	3	2	2.1	0.3	13.0	23
	*L. elegans*	2	3	2	2.1	0.3	15.0	25
Oral basket length (μm)	*P. marina*	11	14	12	12.4	4.3	8.5	25
	*A. cucumis*	7	11	9	9.2	0.9	9.5	25
	*L. minutus*	6	7	7	6.7	0.5	6.8	25
	*L. elegans*	8	11	10	9.5	1.0	10.3	25
Oral basket width (μm)	*P. marina*	9	15	12	12.1	1.7	14.4	25
	*A. cucumis*	3	5	4	4.1	0.6	14.6	25
	*L. minutus*	2	3	2	2.2	0.5	19.4	25
	*L. elegans*	4	6	5	5.1	0.6	13.0	25
Ratio of oral basket length to oral basket width	*P. marina*	0.8	1.3	1	1.1	0.1	11.1	25
	*A. cucumis*	2	3	2	2.3	0.3	12.3	25
	*L. minutus*	2	4	3	3.1	0.5	15.6	25
	*L. elegans*	1	2	2	1.9	0.3	13.9	25
Somatic kineties number	*P. marina*	42	60	50	50.2	4.3	8.5	25
	*A. cucumis*	20	24	22	22.1	1.4	6.1	25
	*L. minutus*	10	11	10	10.5	0.5	4.9	25
	*L. elegans*	26	37	30	30.5	2.9	9.6	25
Perioral kineties number	*P. marina*	7	7	7	7.0	0.0	0.0	25
	*A. cucumis*	3	3	3	3.0	0.0	0.0	25
	*L. minutus*	3	3	3	3.0	0.0	0.0	25
	*L. elegans*	3	3	3	3.0	0.0	0.0	25
Cervical kineties number	*P. marina*	10	14	12	12.1	1.2	10.0	20
	*A. cucumis*	4	7	5	5.4	0.9	16.9	25
	*L. minutus*	4	5	5	4.5	0.5	11.3	25
	*L. elegans*	5	6	6	5.6	0.5	8.7	25

These data are based on stained specimens

*CV* coefficient of variation (%), *M* median, *Max* maximum, *Min* minimum, *n* number of specimens examined, *SD* standard deviation

**Diagnosis** Cell size 160–275 × 20–60 μm in vivo; one sausage-shaped macronucleus and a long ellipsoidal micronucleus; extrusomes rod-shaped, located near the cytostome; contractile vacuole terminal, triangular; rows of oblong protrusions on cell surface present; seven dikinetidal perioral kineties, 10–14 cervical kineties, and 42–60 somatic kineties. Seawater habitat.

**Fig. 1:**
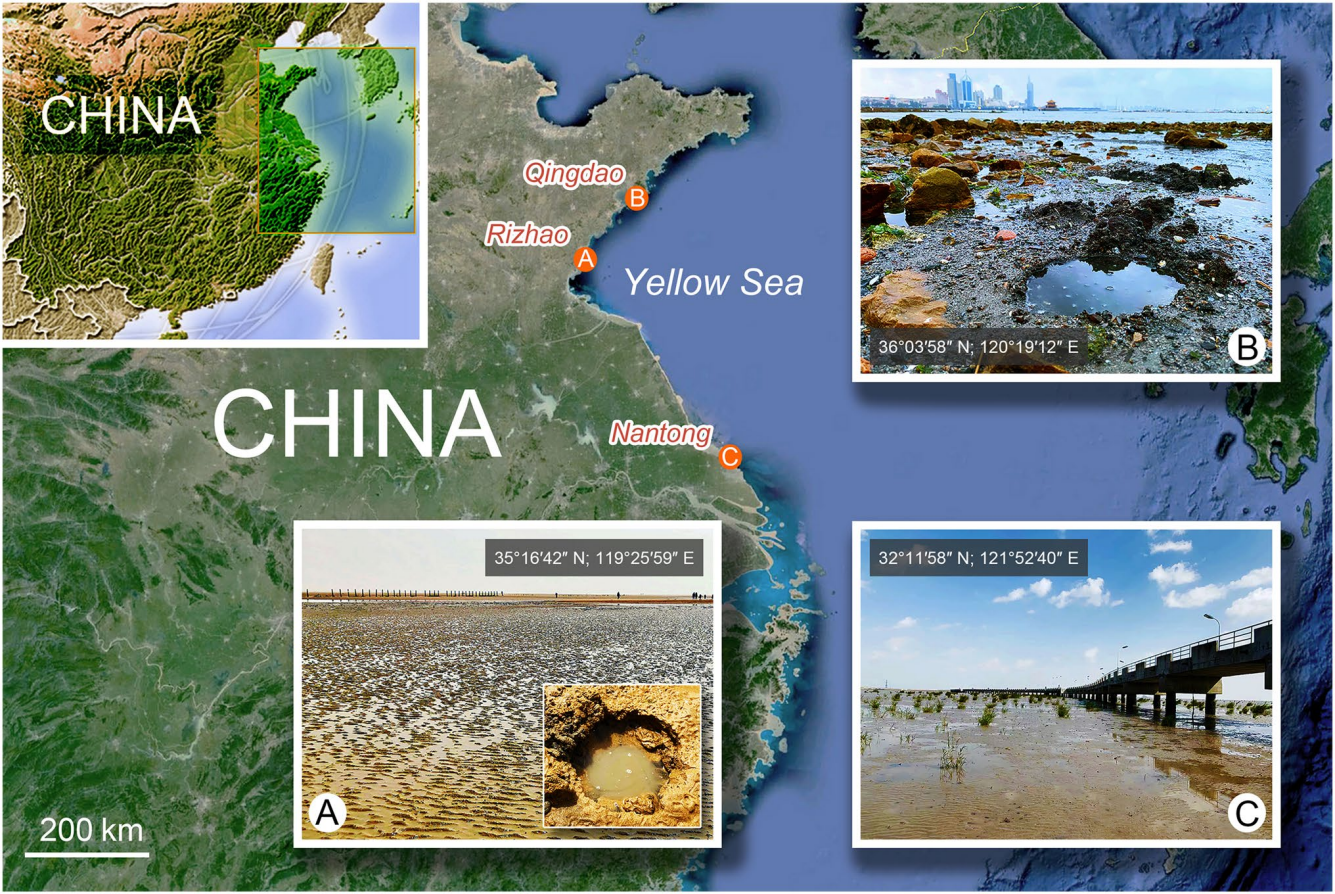
Maps and photographs showing sampling sites investigated during this study. **A** Liujiawan Park where *Penardella marina* gen. nov., sp. nov. was collected. **B** Zhanqiao Pier where *Apolagynus cucumis* gen. nov., comb. nov. and *Lagynus elegans* were collected. **C** Liya Hill where *Lagynus minutus* sp. nov. was collected

**Type locality** This species was found in the intertidal zone of a sandy beach at Liujiawan Park, Rizhao, Shandong Province, northern China (35°16′42″ N; 119°25′59″ E). The salinity was 31 and the water temperature was 24 °C at the time of sampling (Fig. 1).

**Fig. 2:**
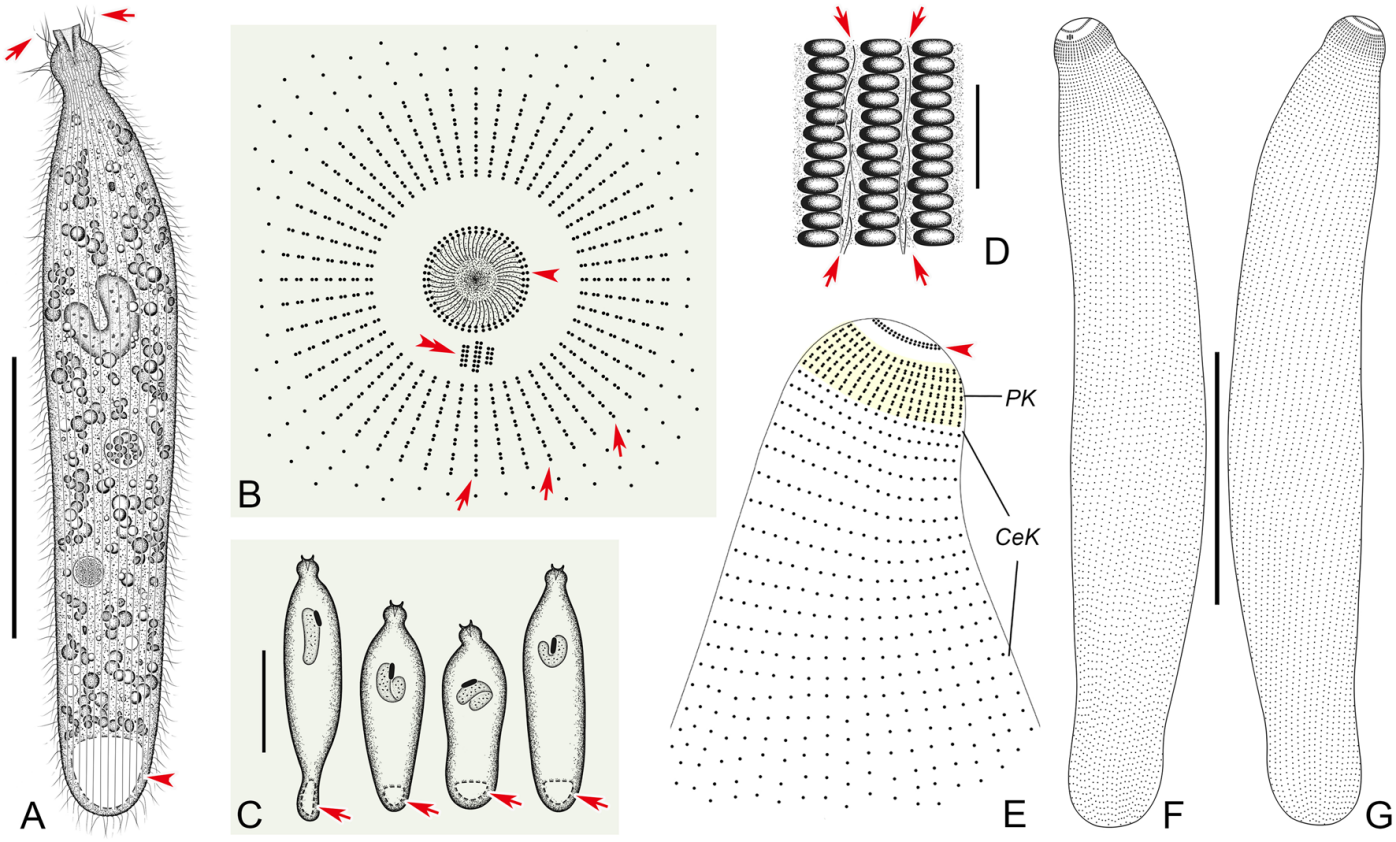
*Penardella marina* gen. nov., sp. nov. from life (**A**, **C**, **D**) and after protargol staining (**B**, **E**–**G**). **A** Lateral view of a typical individual, showing the general appearance of the cell, perioral cilia (arrows) and contractile vacuole (arrowhead). **B** Apical view of the anterior portion of the cell, schematic illustration showing the circumoral kinety (arrowhead), brosse (double arrowhead) and perioral kineties (arrows). **C** Shape variants of the cell and contractile vacuole differences (arrows). **D** Oblong protrusions on cell surface, arrows showing the somatic cilia. **E** Anterior end showing the circumoral kinety (arrowhead), perioral kineties (PK) and cervical kineties (CeK). **F**, **G** General infraciliature of the holotype specimen. *CeK* cervical kineties, *PK* perioral kineties. Scale bars = 90 μm (**A**, **C**), 10 μm (**D**)

**Type specimens** One protargol slide containing the holotype specimen and paratype specimens (Fig. 2F, G) (registration number: JLM2021051301-1) was deposited in the Laboratory of Protozoology, Ocean University of China.

**Etymology** The species name *marina* (Latin adjective; living in the sea) alludes to the marine habitat where the species was found.

**Fig. 3:**
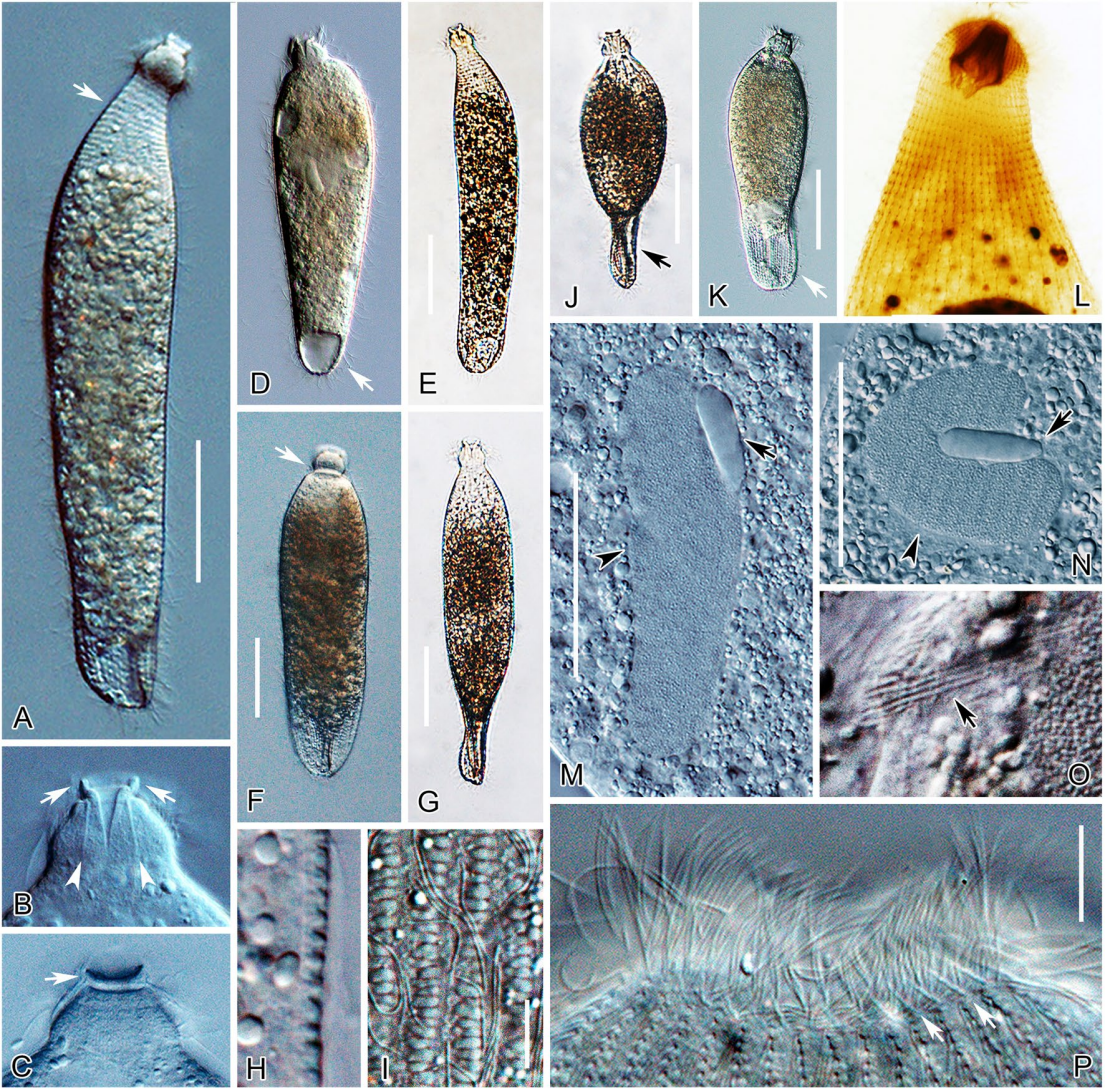
Photomicrographs of *Penardella marina* gen. nov., sp. nov. from life (**A**–**K**, **M**–**P**) and after protargol staining (**L**). **A**, **E** Lateral view of a typical individual, showing the cell shape and furrows around the neck-like region (arrow). **B**, **C** Side view of anterior portion of the cell, showing the nematodesmata (arrowheads) and apical collar (arrows). **D** The contractile vacuole (arrow). **F** Showing the variation in neck shape when contracted (arrow). **G**, **J**, **K** Showing shape variants and the flat posterior end of the cell at different angles relative to the main cell axis (arrows). **H** Lateral view of the oblong protrusions. **I** Oblong protrusions on cell surface. **L** Anterior end showing the infraciliature. **M**, **N** The macronucleus (arrowheads) and micronucleus (arrows). **O** The rod-shaped extrusomes distributed near cytostome (arrow). **P** The perioral cilia (arrows). Scale bars = 50 μm (**A**, **E**–**G**, **J**, **K**, **M**, **N**), 10 μm (**I**, **P**)

**Description** Fully extended cell about 160–275 × 20–60 μm in vivo, mostly 230 × 50 μm, usually elongate cylindroid, progressively narrowed from middle toward both ends; when starved, posterior quarter to third of cell flattened and transparent; length to width ratio highly variable among different individuals, about 4–7:1 (Figs. 2A, C, 3A, D–G, J, K). Cell flexible and contractile, especially in neck-like region; upon contraction, neck retracts and becomes inconspicuous (Fig. 3A, F). Cytostome apically located, circular, with a conspicuous apical protrusion; oral basket trapezoid, about 12 × 12 μm after protargol staining, with visible nematodesmata (about 14 μm long) (Figs. 2A, 3B, C). Deep furrows encircle neck-like region when cell is stationary (Fig. 3A, E). Numerous oblong protrusions on cell surface, arranged in longitudinal rows between adjacent ciliary rows (Figs. 2D, 3H, I). Extrusomes rod-shaped, about 12 μm long in vivo, found only near cytostome (Fig. 3O). Cell generally opaque, full of spherical granules rendering it black-brown (Figs. 2A, 3A, E, J). Single sausage-like macronucleus, usually bent into a “C” shape, positioned about one-third down length of cell, about 90 × 20 μm after protargol staining; single micronucleus, elongated, about 25 × 5 μm in vivo, closely associated with macronucleus (Figs. 2C, 3M, N). Single contractile vacuole, triangular, terminally positioned (Figs. 2A, 3D). Locomotion by swimming while rotating, either clockwise or counterclockwise, about main cell axis.

Most somatic cilia about 8–10 μm long, densely arranged; perioral cilia, about 12–14 μm long (Figs. 2A, 3P). In total 42–60 somatic kineties, each consisting of monokinetids (Figs. 2F, G, E, 3L). Circumoral kinety consists of dikinetids with each pair of kinetosomes vertically aligned (Fig. 2B, E–G). Brosse composed of three rows of narrowly spaced dikinetids; brosse row 1 (B1), brosse row 2 (B2), brosse row 3 (B3) consisting of four, five to seven, and four or five pairs of basal bodies, respectively (Fig. 2B, F, G). Invariably seven perioral kineties, each ring-like and composed of dikinetids (Fig. 2B, E–G). Ten to 14 annular cervical kineties encircle neck-like region, continuous with somatic kineties (Figs. 2E, F, G, 3L).

### Genus: *Apolagynus* gen. nov.

**Diagnosis** Lagynusidae without neck-like region; cortical granules present; three dikinetidal perioral kineties.

**Etymology** The genus name *Apolagynus* is a composite of the Greek adjective *apo-* (away from, off) and the genus name *Lagynus*, alluding that the genus is similar to but different from *Lagynus*. Masculine gender.

**Type species** *Apolagynus binucleatus* (Jiang et al., 2021) comb. nov.

**Species assignable** *Apolagynus binucleatus* (Jiang et al., 2021) comb. nov. (basionym: *Lagynus binucleatus* Jiang et al., 2021); *Apolagynus cucumis* (Penard, 1922) gen. nov., comb. nov. (basionym: *Lacrymaria cucumis* Penard, 1922).

### ***Apolagynus cucumis*** (Penard, 1922) gen. nov., comb. nov. (Fig. 4; Table 1)

**Fig. 4:**
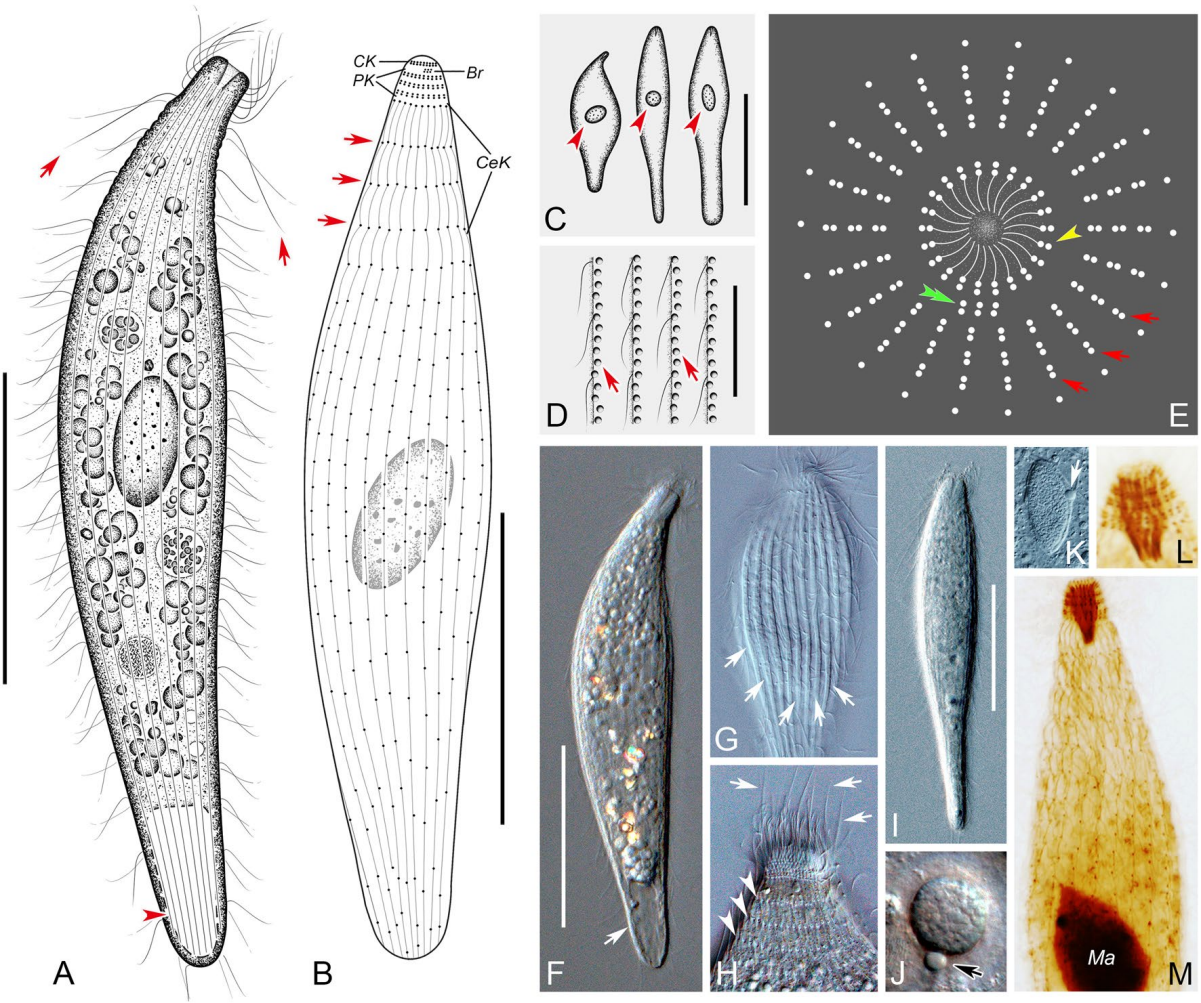
Drawings (**A**–**E**) and photomicrographs (**F**–**M**) of the Chinese population of *Apolagynus cucumis* gen. nov., comb. nov. from life (**A**, **C**, **D**, **F**–**K**) and after protargol staining (**B**, **E**, **L**, **M**). **A** Lateral view of a typical individual, showing the general appearance of cell with elongated cilia (arrows) and contractile vacuole (arrowhead). **B** Side view, showing the ciliary pattern, circumoral kinety (CK), perioral kineties (PK), cervical kineties (CeK) (arrows) and brosse (Br). **C** Showing shape variants and the macronucleus (arrowheads). **D** The distribution of cortical granules (arrows). **E** Apical view of the anterior portion of the cell, schematic illustration showing the circumoral kinety (arrowhead), brosse (double arrowhead) and perioral kineties (arrows). **F** Lateral view of a typical individual, showing the cell shape and contractile vacuole (arrow). **G** Longitudinal ridges on cell surface (arrows). **H** Long cilia distributed in the first ring of the cervical kineties (arrows) and the cervical cilia (arrowheads). **I** Elongated cell variant. **J**, **K** Variations of the macronucleus and micronucleus (arrows). **L** Side view of the oral aperture. **M** Side view, showing the ciliary pattern and macronucleus (Ma). *Br* brosse, *CeK* cervical kineties, *CK* circumoral kinety, *Ma* macronucleus, *PK* perioral kineties. Scale bars = 35 μm (**A**–**C**, **F**, **I**), 6 μm (**D**)

Since the original description (Penard 1922), *Apolagynus cucumis* has been redescribed only once (Kahl 1930). Prior to this study, it has never been investigated using the protargol staining, and molecular sequences were also absent. An improved diagnosis based on the new information and previous reports is presented.

**Improved diagnosis** Cell size 80–200 × 15–25 μm in vivo; generally fusiform; single globular to ellipsoidal macronucleus and one globular micronucleus; cortical granules spherical, less than 0.5 μm in diameter; longitudinal ridges on cell surface; terminal contractile vacuole; four to seven cervical kineties and 20–24 somatic kineties. Seawater habitat.

**Description** Extended cells about 80–115 × 15–25 μm in vivo, 100 × 20 μm on average (n = 25), with a length to width ratio of approximately 4:1 (Fig. 4A, F, I). Cell flexible and slightly contractile, especially in anterior portion. Shape highly variable among different individuals, generally elongate fusiform with posterior end broadly rounded and slightly wider than anterior end (Fig. 4A, C, F, I). Anterior two thirds of cell cylindrical, posterior third usually flattened and transparent (Fig. 4F). Cytostome apical; oral basket conical, about 4 μm wide and 9 μm long after protargol staining; nematodesmata well-developed and conspicuous, each about 18–22 μm long (Fig. 4H, L, M). Several inconspicuous transverse furrows around region below anterior end of cell (Fig. 4A). Cortical granules spherical and colorless, 0.3–0.4 μm in diameter, arranged in longitudinal rows (Fig. 4D). Cell surface sculptured with strongly marked longitudinal ridges (Fig. 4G). Cytoplasm colorless and transparent, packed with numerous food vacuoles and spherical granules (Fig. 4A, F). Single macronucleus globular to ellipsoidal, positioned in mid-cell region, size about 18 × 13 μm after protargol staining; single micronucleus, close to macronucleus, globular, about 3 μm in diameter and easily recognized in vivo (Fig. 4J, K). Single contractile vacuole, caudally positioned, with inconspicuous collecting channels (Fig. 4A, F). Locomotion by rotating about the main cell axis with the anterior end swinging from side to side.

Most somatic cilia about 12 μm long; in total, 20–24 longitudinal somatic kineties composed of monokinetids (Fig. 4B, M). One circumoral kinety consisting of dikinetids, each pair of basal bodies vertically aligned and only one kinetosome of each dikinetid bears a cilium, about 6 μm long (Fig. 4B, E, L). Brosse composed of three pairs of kinetids; slightly inclined at an angle relative to main cell axis (Fig. 4B, E, L). Invariably three perioral kineties, each ring-like and composed of dikinetids, located at anterior ends of somatic kineties (Fig. 4B, E, L). Five to seven cervical kineties, located at the above-mentioned furrows, with cervical cilia arranged in regular rings and about 9 μm long; except for a few extremely long cilia on the first ring, about 25 μm long (Fig. 4A, B, H, M).

### Genus: ***Lagynus*** Quennerstedt, 1867

Here we provide an improved diagnosis that integrates data from live and silver-stained specimens, which were missing from the previous definitions for this genus.

**Improved diagnosis** Lagynusidae with a neck-like region that is slightly contractile and encircled by conspicuous furrow formations; extrusomes present; three dikinetidal perioral kineties.

### ***Lagynus elegans*** (Engelmann, 1862) Quennerstedt, 1867 (Fig. 5; Table 1)

**Fig. 5:**
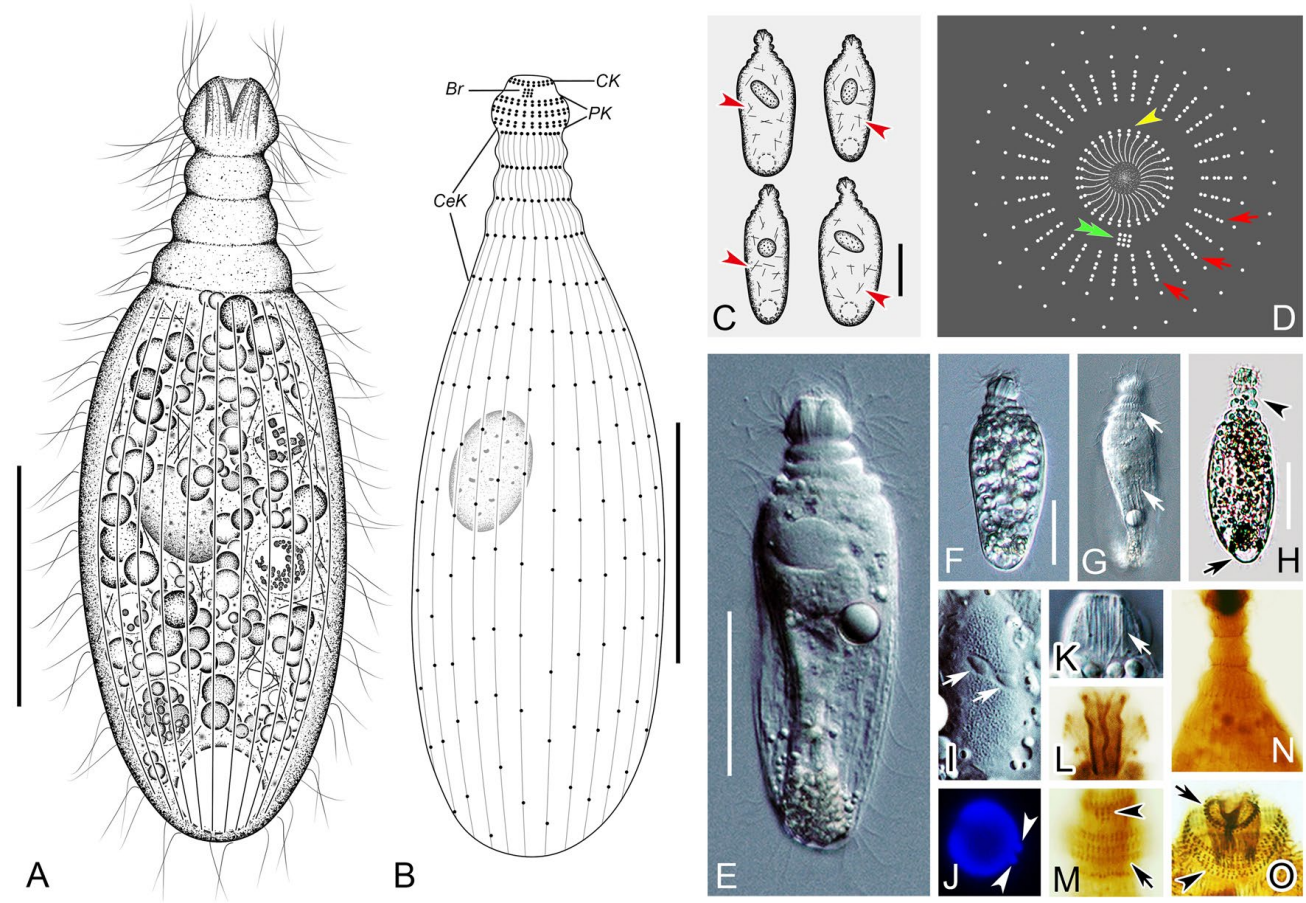
Drawings (**A**–**D**) and photomicrographs (**E**–**O**) of the Chinese population of *Lagynus elegans* from life (**A**, **C**, **E**–**I**, **K**), after DAPI staining (**J**), and after protargol staining (**B**, **D**, **L**–**O**). **A** Lateral view of a typical individual, showing the general appearance of the cell. **B** Side view, showing the ciliary pattern, circumoral kinety (CK), perioral kineties (PK), cervical kineties (CeK) and brosse (Br). **C** Showing shape variants and extrusomes (arrowheads). **D** Apical view of the anterior portion of the cell, schematic illustration showing the circumoral kinety (arrowhead), brosse (double arrowhead) and perioral kineties (arrows). **E**, **F** Starved cells. **G** The longitudinal ridges on starved cell surface (arrows). **H** Lateral view of a typical individual, showing the cell shape, contractile vacuole (arrow) and retractable neck-like region (arrowhead). **I** The macronucleus and two micronuclei (arrows). **J** The macronucleus and two micronuclei (arrowheads). **K** Showing the nematodesmata (arrow). **L** The oral basket (dark ‘V’ at center). **M** Ciliary pattern of the anterior portion of the cell, showing the brosse (arrowhead) and perioral kineties (arrow). **N** The elongated neck showing the cervical kineties (CeK). **O** Showing the circumoral kinety (arrow) and perioral kineties (arrowhead). *Br* brosse, *CeK* cervical kineties, *CK* circumoral kinety, *PK* perioral kineties. Scale bars = 35 μm

This species was originally reported by Engelmann (1862) and was subsequently redescribed several times (Foissner et al. 1995; Kahl 1930; Liebmann 1962; Penard 1922; Quennerstedt 1867; Sola et al. 1990; Wetzel 1928). Based on new data from the Chinese population, it is redefined as follows.

**Improved diagnosis** Cell size 80–200 × 30–60 μm in vivo; amphora-shaped; single globular to long-ellipsoidal macronucleus and one or two micronuclei; rod-shaped extrusomes, irregularly distributed beneath pellicle; contractile vacuole terminally located; three to six cervical kineties and 26–50 somatic kineties. Freshwater and brackish water habitat.

**Description** Cell size in vivo about 80–125 × 30–55 μm, 100 × 30 μm on average (n = 25), with a length to width ratio of 3–4:1 (Fig. 5A, C, E, F, H). Cell generally amphora-shaped, with an enlarged ellipsoidal main part, posterior end broadly rounded; starved individuals cylindroid, with posterior half of cell flattened and transparent (Fig. 5A, C, E, F, H). Neck-like region flexible and contractile, with four deep furrows (Fig. 5A, E, H). Oral bulge prominently projected from anterior end of cell, forming an apical plate; cytostome apical and circular; oral basket inverted trapezoidal, size about 8–11 × 4–6 μm after protargol staining; rod-shaped nematodesmata about 7 μm long in vivo (Fig. 5A, E, K, L, O). Cell surface with longitudinal ridges throughout cell length (Fig. 5G). Cytoplasm opaque and grayish due to being filled with multitudinous food vacuoles and light-refracting granules (Fig. 5A, F, H). Extrusomes 6–10 μm long in vivo, rod-shaped, irregularly distributed beneath pellicle (Fig. 5C). One macronucleus, globular to long-ellipsoidal, located in mid-cell, size about 18–33 × 8–17 μm after staining; two globular to ellipsoidal micronuclei, closely associated with macronucleus, size about 1–2 × 3–4 μm after DAPI staining (Fig. 5I, J). Single contractile vacuole, terminally located, about 11 μm in diameter (Fig. 5A, H). Locomotion by swimming moderately fast, with no obvious pattern.

Somatic cilia about 10 μm long in vivo; in total, 26–37 somatic kineties consisting of monokinetids (Fig. 5B, N). One dikinetidal circumoral kinety encircling anterior end of cell (Fig. 5B, D, O). Brosse region composed of three rows of kineties, each of which contains two or three basal bodies (Fig. 5B, D, M). Invariably three perioral kineties, each ring-like and composed of dikinetids (Fig. 5B, D, M, O). Five or six cervical kineties around neck-like region, arranged in rings (Fig. 5B, N).

### ***Lagynus minutus*** sp. nov. (Fig. 6; Table 1)

**Fig. 6:**
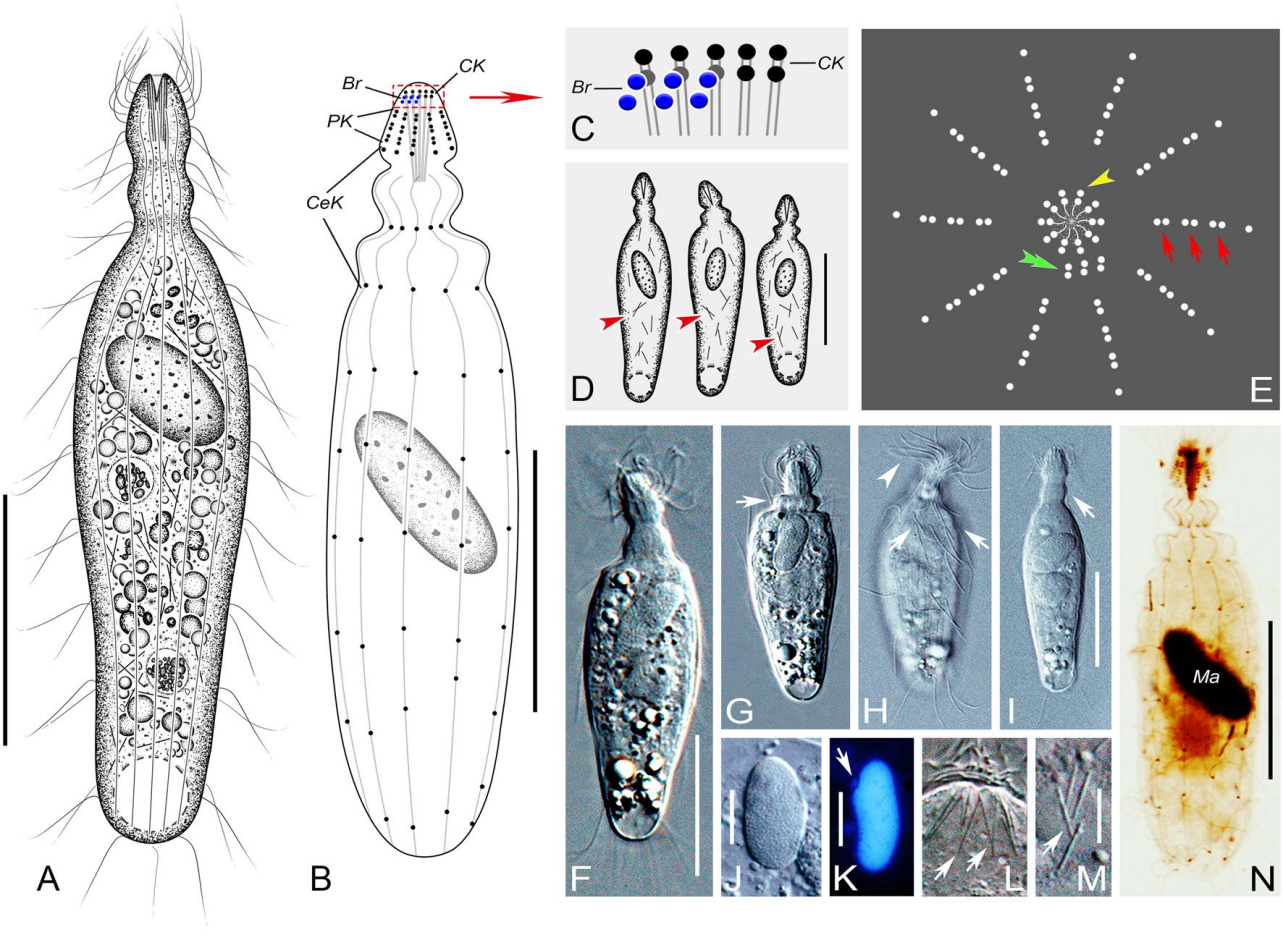
Drawings (**A**–**E**) and photomicrographs (**F**–**N**) of *Lagynus minutus* sp. nov. from life (**A**, **D**, **F**–**J**, **L**, **M**), after DAPI staining (**K**), and after protargol staining (**B**, **C**, **E**, **N**). **A** Lateral view of a typical individual, showing the cell shape. **B** Lateral view of the holotype specimen, showing the ciliary pattern, circumoral kinety (CK), perioral kineties (PK), cervical kineties (CeK) and brosse (Br). **C** The brosse (Br) and circumoral kinety (CK). **D** Showing shape variants and extrusomes (arrowheads). **E** Apical view of the anterior portion of the cell, schematic illustration showing the circumoral kinety (arrowhead), brosse (double arrowhead) and perioral kineties (arrows). **F**, **G** Lateral view of slightly compressed individuals, showing the neck-like region after contraction (arrow). **H** The perioral cilia (arrowhead) and cervical cilia (arrows). **I** Lateral view of a typical individual, showing the cell shape and neck-like region (arrow). **J** The macronucleus. **K** The macronucleus and micronucleus (arrow). **L** Showing the nematodesmata (arrows). **M** Showing the extrusomes (arrow). **N** Lateral view of the holotype specimen, showing ciliary pattern and macronucleus (Ma). *Br* brosse, *CeK* cervical kineties, *CK* circumoral kinety, *Ma* macronucleus, *PK* perioral kineties. Scale bars = 20 μm (**A**, **B**, **D**, **F**, **I**, **N**), 5 μm (**J**, **K**, **M**)

**Diagnosis** Cell size 45–65 × 10–25 μm in vivo; amphora shaped; one ellipsoidal macronucleus and single ellipsoidal micronucleus; extrusome rod-shaped, irregularly distributed beneath pellicle; contractile vacuole terminally positioned, globular; four or five cervical kineties and 10–11 somatic kineties. Seawater habitat.

**Type locality** This species was found in the intertidal zone of a sandy marine beach at Liya Hill, Nantong, Jiangsu Province, northern China (32°11′58″ N; 121°52′40″ E). The salinity was 24 and the water temperature was 21 °C at the time of sampling.

**Type specimens** One protargol slide that contains the holotype specimen and paratype specimens (Fig. 6B, N) (registration number: JLM2021051001-1) was deposited in the Laboratory of Protozoology, Ocean University of China.

**Etymology** The species name *minutus* (Latin adjective, meaning small, tiny) refers to its small size.

**Description** Cell size in vivo about 45–65 × 10–25 μm, 50 × 20 μm on average (n = 25), with a length to width ratio of 3–4:1; cell generally amphora-shaped with a cylindroid main part, gradually narrowed from middle toward both ends; “head” noticeably bulging and wider than neck-like region (Fig. 6A, D, F, G, I). Body flexible but not contractile, except for neck-like region which is obviously retractable (Fig. 6A, F, G, I). Cytostome apical and inconspicuous; oral basket slender and conical, about 6–7 μm long and 2–3 μm wide at anterior end after protargol staining; rod-shaped nematodesmata near cytostome, about 7 μm long in vivo (Fig. 6A, L, N). Longitudinal continuous shallow grooves present on cell surface, regularly arranged in two rows between each pair of adjacent kineties (Fig. 6H). Cytoplasm transparent and colorless, containing several spherical light-refracting granules (Fig. 6A, F, G, I). Extrusomes rod-shaped, about 4–5 μm long in vivo, randomly distributed beneath pellicle (Fig. 6D, M). One ellipsoidal to long-ellipsoidal macronucleus, centrally located, about 8–15 × 4–7 μm in size after protargol staining; closely associated it is the ellipsoidal micronucleus, size about 1 × 2 μm after DAPI staining (Fig. 6J, K). Single contractile vacuole, caudally located, about 10 μm in diameter when fully expanded (Fig. 6A, F). Locomotion by swimming moderately fast in upper layer of water, without fixed pattern.

Somatic cilia about 13 μm long; in total, 10–11 monokinetid somatic kineties, sparsely arranged (Fig. 6B, E, N). Circumoral kinety composed of dikinetids, each pair of kinetosomes aligned along direction of oral basket (Fig. 6B, C, E). Brosse located on lateral side of circumoral kinety, composed of three pairs of kinetids and parallel to main cell axis (Fig. 6B, C, E). Invariably three perioral kineties, each ring-like and composed of dikinetids (Fig. 6B, E). Four or five cervical kineties, with cervical cilia arranged in regular rings (Fig. 6B, N).

### Molecular data and phylogenetic analyses

The new 18S rRNA gene sequences were deposited in the GenBank database with the following accession numbers, lengths, and guanine–cytosine (GC) content: *Penardella marina* gen. nov., sp. nov. (OP292226, 1617 bp, 45.33%), *Apolagynus cucumis* gen. nov., comb. nov. (OP292227, 1597 bp, 45.34%), *Lagynus minutus* sp. nov. (OP292225, 1628 bp, 44.41%), and *Lagynus elegans* (OP292224, 1640 bp, 43.54%).

**Fig. 7:**
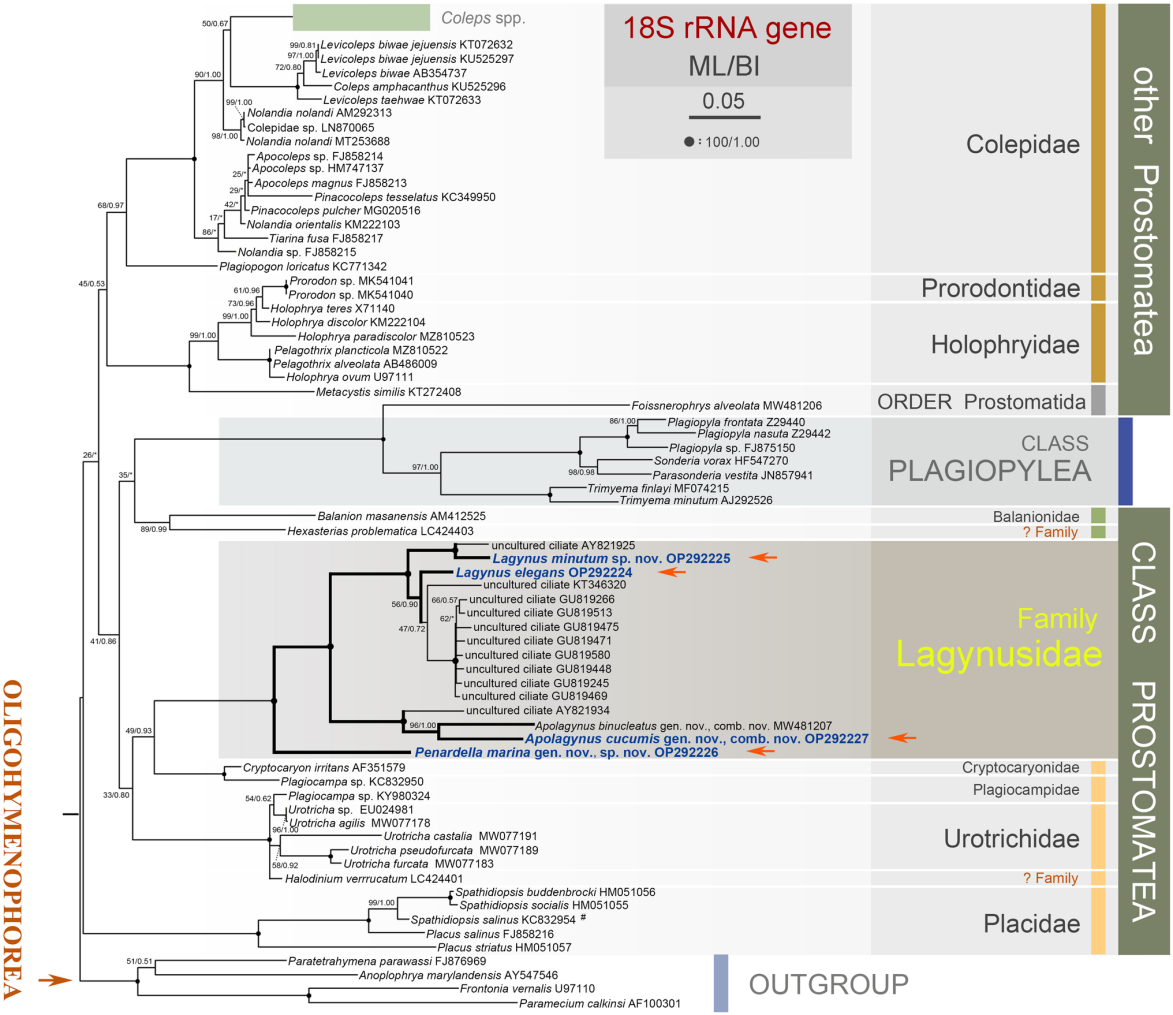
Maximum likelihood tree inferred from 18S rRNA gene sequences, showing the phylogenetic positions of the four newly sequenced species (bold blue font). Numbers near the nodes represent the maximum likelihood (ML) bootstrap values and Bayesian inference (BI) posterior probabilities, respectively. Fully supported (100/1.00) branches are marked with solid circles. Asterisks (*) indicate disagreements between the ML and BI trees. The number sign (#) indicates misidentification as *Placus salinus* in the GenBank database, where it is should be named as *Spathidiopsis salinus*. The scale bar corresponds to five substitutions per 100 nucleotide positions. Morphological characters of lagynusid species are marked with vertical bars, colored according to the inset key

Maximum likelihood (ML) and Bayesian inference (BI) trees from this study have similar topologies, therefore only the ML tree is presented here with support values from both analyses (Fig. 7). Members of the family Placidae cluster together with full support (ML/BI, 100/1.00), forming a monophyletic group and the basal branch of the class Prostomatea. The remaining families of the Prostomatea are all grouped together with species of the class Plagiopylea nested within it. The family Lagynusidae is monophyletic and is closely related to *Cryptocaryon*, *Plagiocampa*, *Urotricha*, and *Halodinium*; and above groups form a subclade with weak support (ML/BI, 33/0.80). All species of Lagynusidae form a maximally supported clade (ML/BI, 100/1.00), with *Penardella marina* occupying the basal position. *Apolagynus* species (*A. binucleatus* comb. nov., *A. cucumis* and an uncultured species) form a robust clade (ML/BI, 100/1.00) that is a sister to a maximally supported clade (ML/BI, 100/1.00) comprising *Lagynus* species (*L. elegans*, *L. minutus* and some unidentified species). The two above-mentioned clades form a fully supported clade (ML/BI, 100/1.00) that is clustered with the *Penardella marina*.

## Discussion

### Comments on the family Lagynusidae Sola et al., 1990 nom. emend.

The ciliate family-group name Lagynidae Sola et al., 1990 is a junior homonym of the foraminiferan family-group name Lagynidae Schultze, 1854 (ICZN 1999). However, they are based on non-homonymous generic names, viz. *Lagynus* Quennersted, 1867 and *Lagynis* Schultze, 1854. According to Articles 55.3.1 and 29 of “the code” (ICZN 1999), we propose an emendation of the junior name, using the full generic name as the stem (hence “Lagynusidae”), to remove the homonymy.

Prior to this study, *Lagynus* was the only genus of the family Lagynusidae. It contained three species, namely *Lagynus elegans* (Engelmann, 1862) Quennerstedt, 1867, *Lagynus cucumis* (Penard, 1922) Foissner, 1987, and *Lagynus binucleatus* Jiang et al., 2021. It is noteworthy that when the genus *Lagynus* was established, it accommodated those species of *Lacrymaria* that are only slightly contractile (Quennerstedt 1867). However, these species were transferred to the genera *Lacrymaria*, *Enchelys*, *Enchelyodon*, and *Trachelocerca* by Kahl (1930). The taxonomic history of Lagynusidae had been provided previously by Jiang et al. (2021b). In general, *Lagynus* inhabits aquatic zones associated with oxygen-depleted sediments and is therefore an anaerobic ciliate (Finlay et al. 1991), with the species examined here likely also inhabiting this niche.

In the present work, two new lagynusids, *Lagynus minutus* sp. nov. and *Penardella marina* gen. nov., sp. nov., are reported and the new genus *Penardella* is established. In addition, we redescribe in detail two known species, viz. *Lagynus elegans* and *Lagynus cucumis*. After a detailed comparison of the above species, we establish the new genus *Apolagynus* to accommodate *Apolagynus cucumis* gen. nov., comb. nov. and *Apolagynus binucleatus* comb. nov. Based on this new information, a user-friendly identification to five known lagynusids is supplied below.

Key to the known species in the family Lagynusidae.

1. Three perioral kineties………….............…..........................….…2

More than three perioral kineties…………………............................*Penardella marina* gen. nov., sp. nov.

2. Extrusomes and neck-like region absent……………..........………3

Extrusomes and neck-like region present.....………........……………4

3. Single macronucleus………………..........…*Apolagynus binucleatus* gen. nov., comb. nov.

Two macronuclear nodules………………..……*Apolagynus cucumis* gen. nov., comb. nov.

4. One neck furrow encircling the neck-like region………………......…*Lagynus minutus* sp. nov.

More than one furrow encircling the neck-like region……………………*Lagynus elegans.*

### Comments on the genus *Penardella* gen. nov.

Based on the elongated cell shape, annular neck-like region, and circular perioral kineties, *Penardella marina* gen. nov., sp. nov. should be assigned to the family Lagynusidae (Foissner et al. 1995; Jiang et al. 2021b; Sola et al. 1990). However, *Penardella marina* gen. nov., sp. nov. differs from other lagynusid species by its large clavate (vs. amphora-shaped or fusiform) cell shape, sausage-like (vs. globular or ellipsoidal) macronucleus, elongate (vs. globular to ellipsoidal) micronucleus, and the presence (vs. absence) of rows of oblong protrusions on the cell surface (Table 3). Furthermore, the most conspicuous and diagnostically important feature that distinguishes this new species from other lagynusids is the number of perioral kineties (seven vs. three in *Lagynus* and *Apolagynus*) (Foissner et al. 1995; Jiang et al. 2021b; Sola et al. 1990) (Table 3). This suggests that a new genus, *Penardella* gen. nov., should be established to accommodate this unique species.

### Comments on the genus *Apolagynus* gen. nov.

Although the pattern of infraciliature of *Apolagynus* gen. nov*.* is almost identical to that of the genus *Lagynus*, the new genus can be easily distinguished from the latter via numerous characteristics observed in vivo: the former exhibits an elongate-fusiform (vs. ellipsoidal to cylindroid) cell shape, the presence (vs. absence) of spherical cortical granules arranged in longitudinal rows, and the absence (vs. presence) of a neck-like region or extrusomes (Fig. 11). Since these characters are diagnostic and group-related, *Apolagynus* should be separated from *Lagynus* (Foissner et al. 1995; Kahl 1930; Quennerstedt 1867; Sola et al. 1990).

### Comments on ***Apolagynus cucumis*** (Penard, 1922) gen. nov., comb. nov.

As a little-known form, this species has been previously described only twice. Penard (1922) first reported it as *Lacrymaria cucumis*. Later, Kahl (1930) discovered this species in freshwater pools and humus-rich saltworks of Germany. This species was later transferred to the genus *Lagynus* due to its inconspicuous neck-like region (Foissner 1987).

The Chinese population matches the Swiss population well in terms of the diagnostic characteristics. Both populations possess an elongate fusiform cell body, single macronucleus, transverse ciliary rings at the anterior of the cell (corresponding to the perioral kineties), strongly marked longitudinal ridges on the cell surface, and a large, caudally positioned contractile vacuole with accompanying collecting channels. Although the specimens of Penard (1922) are slightly larger sized (100–190 μm vs. 80–115 μm in length) than the population investigated here, the two populations are likely conspecific. Cell sizes of ciliates can be environmentally dependent as shown by reports of a range in sizes among different geographic populations within the same species (Dragesco 1960; Hartwig 1977; Jiang et al. 2021a; Lipscomb and Riordan 1991; Xu et al. 2011; Yan et al. 2013, 2016). The specimens reported by Kahl (1930) also match the two above mentioned populations in terms of the slender cell, the single macronucleus and micronucleus, the large contractile vacuole, the elongate neck-like region, and the presence of longitudinal ridges on the cell surface. Kahl (1930) further described the delicate nematodesmata near the cytostome, which corresponds well with the Chinese population.

According to the finding presented here, a recently discovered species,* Lagynus binucleatus* Jiang et al., 2021, possesses the diagnostic characters of *Apolagynus*, and should therefore be transferred to this new genus. It is necessary to correct a misinterpretation (within Jiang et al. 2021b) about the perioral kineties and cervical kineties, which were described as follows: “four perioral kineties, three anterior rows composed of dikinetids, with the posterior-most row composed of monokinetids; and 8–14 cervical kineties” (Jiang et al. 2021b). In fact, the posterior-most monokinetidal row of perioral kineties is a cervical kinety, so we correct the species diagnosis as follows: three perioral kineties composed of three rings of dikinetids; 9–15 cervical kineties (Table 3).

### Comments on the genus ***Lagynus*** Quennerstedt, 1867

The type species *Lagynus elegans* was first discovered in Germany by Engelmann (1862) who described it under the name *Lacrymaria elegans*. Quennerstedt (1867) transferred it to the new genus *Lagynus* as the type species. Subsequently, the species was redescribed several times under the name *Lagynus elegans* (e.g., Penard 1922; Wetzel 1928). However, Kahl (1930) did not accept the classification of this species, so he maintained the original name of *Lacrymaria elegans*. When Sola et al. (1990) first supplied the details of the infraciliature, the taxonomic status of this species was amended, and the new family Lagynusidae was established for *Lagynus*. A later redescription further revised the diagnostic characteristics of this species, supporting its taxonomic status (Foissner et al. 1995).

The Chinese population corresponds well to the original description regarding most of its morphological features, that is, the amphora-shaped cell, the annular neck-like region, the single macronucleus, the longitudinal ridges on the cell surface, and the flat posterior half of cell, although the cell size is smaller (80–125 μm vs. 160–170 μm in length) than that of the original population (Engelmann 1862) (Table 2). Four other populations of *L. elegans* have been reported, but without details of the infraciliature, with the Chinese population closely matching these in terms of the main diagnostic features (i.e., cell shape and size, single macronucleus, annular neck-like region) (Kahl 1930; Liebmann 1962; Penard 1922; Wetzel 1928) (Table 2). Compared to the population described by Sola et al. (1990), the Chinese population exhibits a similar cell size (80–125 μm vs. 75–116 μm in length) but fewer somatic kineties (26–37, 30 on average vs. 37–46) (Table 2). Foissner et al. (1995) reported the distribution of extrusomes within the cytoplasm of the Austrian population, which matches well with our observations (Table 2). However, only one micronucleus was recorded in all the above populations, whereas the Chinese population clearly shows two micronuclei. It is noteworthy that the size difference of the micronucleus between the two populations observed by Penard (1922) was significant although there was only one micronucleus per cell in each population. In addition, a variable number of micronuclei was also reported within the Chinese population of the prostomatean *Pelagothrix plancticola* Foissner et al., 1999 (Jiang et al. 2022). Thus, the number of micronuclei may be variable within *Lagynus elegans*, and could be a population-level difference.

**Table 2:** Comparison of Qingdao population of *Lagynus elegans* with other populations

Population	Germany pop. I	UK pop.	Switzerland pop.	Germany pop. II	Germany pop. III	Germany pop. IV	Spain pop.	China pop.
Habitat	Freshwater	Freshwater	Freshwater	Freshwater	–	Freshwater	Freshwater	Brackish water (salinity 6)
Cell length in vivo (μm)	160–170	170–200	125–150	150–160	130–200	70–160	75–116	80–125
Cell shape	Amphora shaped	Flask–shaped	Cylindrical	Globular to long cylindrical	Bag–shaped, cylindrical	Long cylindrical	Amphora-shaped	Amphora-shaped
Ma number	1	1	1	1	1	1	1	1
Ma shape	Ellipsoidal	Oval	Ellipsoidal	Globular, ellipsoidal, kidney–shaped	Ellipsoidal, kidney–shaped	Ellipsoidal, kidney–shaped	Ellipsoidal, kidney–shaped	Globular to long ellipsoidal
Mi number	1	–	1	–	–	–	1	2
Mi shape	Globular	–	Ellipsoidal	–	–	–	Ellipsoidal	Globular to ellipsoidal
Number of neck rings	4–5	3–4	3	3–5	4–5	3–5	4–5	4
Presence of longitudinal ridges	Yes	Yes	–	Yes	–	Yes	–	Yes
Presence of flat back end	Yes	–	–	Yes	–	Yes	Yes	Yes
Presence of Extrusomes	–	–	Yes	–	–	–	–	Yes
Brosse rows number	–	–	–	–	–	–	3 or 4	3
SK number	–	–	–	About 50	–	About 50	37–46	26–37
Data source	Engelmann (1862)	Kent (1882)	Penard (1922)	Wetzel (1928)	Kahl (1930)	Liebmann (1962)	Sola et al. (1990)	Present study

These data are based on stained specimens (except when specified)

*Ma* macronucleus, *Mi* micronucleus (or micronuclei), *SK* somatic kineties, “–” data unavailable

*Lagynus minutus* sp. nov. should be assigned to the genus *Lagynus* based on its annular neck-like region, rod-shaped extrusomes, and infraciliature (especially the pattern of perioral kineties and brosse). The new species can be easily separated from its congener, *Lagynus elegans*, both in overall appearance and infraciliature (Foissner et al. 1995; Sola et al. 1990) (Table 3). The new species is long cylindroid (vs. with a bulging ellipsoidal main part), is smaller (45–65 μm vs. 80–200 μm in length), has fewer furrows in the neck-like region (2 vs. 4) and substantially fewer somatic kineties (10–11 vs. 26–50) than *L. elegans*. Thus, these two species are clearly separated.

**Table 3:** Comparison of five species within the family Lagynusidae

Characters	*Penardella marina*	*Apolagynus cucumis*	*Apolagynus binucleatus*	*Lagynus minutus*	*Lagynus elegans*
Cell size in vivo (μm)	160–275 × 20–60	80–200 × 15–25	165–340 × 20–60	45–65 × 10–25	80–200 × 30–60
Cell shape	Cylindroid	Fusiform	Spindle- shaped	Amphora-shaped	Amphora-shaped
Oral basket size (μm)	12 × 12	7–11 × 3–5	8–11 × 3–6	6–7 × 2–3	8–11 × 4–6
Extrusomes length (μm)	12	–	–	4–5	6–10
Ma number	1	1	2	1	1
Ma shape	Sausage-like	Globular, ellipsoidal, kidney-shaped	Ellipsoidal	Ellipsoidal to long ellipsoidal	Globular, ellipsoidal, reniform
Mi number	1	1	1	1	1–2
Mi shape	Long ellipsoidal	Globular to ellipsoidal	Ellipsoidal	Ellipsoidal	Globular to ellipsoidal
Brosse rows number	3	3	3	3	3 or 4
SK number	42–60	20–24	28–38	10–11	26–50
Perioral kineties number	7	3	3	3	3
Cervical kineties number	10–14	4–7	9–15	4–5	3–6
Nematodesmata length (μm)	14	18–22	21–38	7	7
Habitat	Seawater	Seawater; freshwater	Freshwater	Seawater	Seawater; freshwater
Data source	Present study	Present study; Penard (1922); Kahl (1930)	Jiang et al. (2021b)	Present study	Present study; Engelmann (1862); Sola et al. (1990); Foissner et al. (1995)

These data are based on stained specimens (except when specified)

*Ma* macronucleus (or macronuclear nodules), *Mi* micronucleus (or micronuclei), *SK* somatic kineties, ‘–’ data unavailable

### Phylogenetic analyses

As shown in previous and present molecular phylogenetic studies, the class Prostomatea is not monophyletic due to species of Plagiopylea nesting within it (Fig. 7) (Gao et al. 2016; Jiang et al. 2021b, 2022; Zhang et al. 2014). The relationships of Colepidae, Lagynusidae, Prorodontidae, Holophryidae and Placidae are relatively stable in recent studies, whereas the other families are not well resolved, as indicated by the low support values and the lack of sequences for these taxa both in previous and present analyses (Jiang et al. 2021b, 2022; Yi et al. 2010; Zhang et al. 2014). Here, we focus on evolutionary relationships of the family Lagynusidae (Fig. 7).

Based on the present phylogenetic analyses, lagynusids show a relatively close relationship with species of Cryptocaryonidae, Plagiocampidae, and Urotrichidae, which is consistent with the findings of Jiang et al. (2021b). Plagiocampidae appears to be non-monophyletic, with sequences needing to be obtained from these groups in future studies to provide further clarity. The evolutionary relationships between lagynusids are still not well resolved as indicated by the low support values and the limited number of sequences both in previous studies and in this analysis.

Sola et al. (1990) proposed the establishment of a new family, Lagynusidae, for this unique group based on the type species *Lagynus elegans*. Prior to our study, only Jiang et al. (2021b) have analyzed the phylogenetic relationships of this family. However, because they were limited to a single available sequence, details of its relationships could not be determined. The present phylogenetic analyses based on molecular data presented here support the validity of newly proposed characterizations. Five known lagynusids, including the four newly obtained sequences presented here, clustered together with full statistical support, which suggests that the family Lagynusidae is monophyletic. The close molecular relationship among lagynusids is supported by their morphological similarities, i.e., an elongated cell, circular perioral kineties, and annular neck-like region.

In this study, species of *Apolagynus* and *Lagynus* were separated into two subclades with maximal support, which strongly supports the establishment of the new genus, *Apolagynus*. The morphological characters used for species circumscription and identification of these two genera, e.g., the conspicuous vs. inconspicuous neck-like region, and the presence vs. absence of extrusomes and cortical granules, are consistent with the topology of the 18S rRNA gene tree. *Penardella marina* occupies the basal position within the Lagynusidae and is thus separated from other lagynusids. The position of *Penardella marina* indicates that the number of perioral kineties is a more phylogenetically informative characters than the morphology of the neck-like region (conspicuous vs. inconspicuous) or the presence vs. absence of extrusomes and cortical granules, and supports the establishment of *Penardella* based on molecular data. Future studies from similar habitats from geographically separate regions may reveal further diversity within this interesting group of ciliates. Detailed morphometrics along with molecular sequences will allow for the comparison of the Chinese species to other populations from diverse biogeographic areas such as Europe, the region in which many of the type localities are located, but for which molecular sequencing remains elusive, could be a productive area of focus for future investigations.

## Materials and methods

### Collection and morphological studies

All four species were collected from sandy beaches in China; *Penardella marina* gen. nov., sp. nov. was collected from Rizhao, Shandong Province (35°16′42″ N; 119°25′59″ E) on May 13, 2021, with water metadata recorded as salinity 31 and temperature of 24 °C at the time of sampling (Fig. 1A). *Apolagynus cucumis* gen. nov., comb. nov. and *Lagynus elegans* were collected from Qingdao, Shandong Province (36°03′58″ N; 120°19′12″ E) on May 28, 2021 and November 30, 2020, respectively. Water metadata was recorded as salinity 31 (open sandy beach) and 6 (near a sewage outlet), respectively, and temperature 25 °C and 11 °C, respectively at the time of sampling (Fig. 1B). *Lagynus minutus* sp. nov. was collected from Nantong, Jiangsu Province (32°11′58″ N; 121°52′40″ E) on May 10, 2021, with water metadata recorded as salinity 24 and temperature 21 °C at the time of sampling (Fig. 1C). The salinity and temperature were determined in situ using a portable photometer (YSI 9500, Xylem). Water samples containing sand and sediment were collected using plastic bottles after gently mixing with water. Samples were kept in 2.5 L plastic anaerobic jars with oxygen-scavenging chemicals added as per the manufacturer’s instructions (Thermo Scientific Oxoid AnaeroGen) for one to two weeks at room temperature (approximately 24 °C). Rice grains were added to facilitate the growth of bacterial food for the ciliates. The extraction of ciliates followed the filtration method described by Carey (1992), and the mesh size utilized was 50 μm. Extracted ciliates were picked up with the pipettes from filtered field samples. Starved cells were observed for microscopy a few days after filtration.

Differential interference contrast and bright field microscopy (ZEISS, AXIO Imager D2) were used for the observation and measurement of living ciliates at 400–1000 × magnifications. The ciliature was revealed by the protargol staining method. The nuclear apparatus was showed by the DAPI staining using Hoechst 33,342 (Thermo Fisher Scientific) solution. Measurements and enumeration of stained specimens were performed with 1000 × magnification using a ZEISS microscope, with photomicrographs produced with a ZEISS camera (Axiocam 506 color). Seventeen to 25 individuals were used for counting, measuring and morphometrics for each species. Drawings were made using tracing paper and adjusted by photoshop from photomicrographs. Classification and terminology were mainly according to Lynn (2008), Foissner et al. (1995) and Jiang et al. (2021b).

### Amplification of DNA and phylogenetic analysis

Cell isolation and genomic DNA extraction were conducted mainly according to Jiang et al. (2021a). The 18S rRNA gene was amplified using the primers given by Medlin et al. (1988). Q5 Hot Start High-Fidelity DNA Polymerase (New England BioLabs, MA, USA) was employed to minimize amplification errors (Liu et al. 2022). The relevant parameters of the polymerase chain reaction (PCR) experiments were according to Jiang et al. (2022). The PCR products were sequenced bidirectionally by Tsingke Biological Technology Company (Qingdao, China) (Li et al. 2022). Seqman V. 7.1.0 was used to assemble the contigs (DNAStar).

Newly obtained sequences were aligned with 76 other available sequences downloaded from the NCBI GenBank database using the MUSCLE program on the European Bioinformatics Institute web server (http://www.ebi.ac.uk/Tools/msa/muscle/) (Edgar 2004). Four oligohymenophoreans, namely *Frontonia vernalis* (U97110), *Paramecium calkinsi* (AF100301), *Paratetrahymena parawassi* (FJ876969) and *Anoplophrya marylandensis* (AY547546), were chosen as the outgroup taxa. The resulting alignments were refined by removing the primer sequences at both ends using BIOEDIT v.7.0.5 (Hall 1999). The final alignment included 80 taxa with 1821 positions. Bayesian inference (BI) and maximum likelihood (ML) analyses were carried out according to Jiang et al. (2022). BI analysis was performed with MrBayes v.3.2.7 on XSEDE (Ronquist et al. 2012), using the GTR + I + G model selected by MrModeltest v.2.2 according to the Akaike Information Criterion (Nylander 2004). ML analysis was carried out using RAxML-HPC2 on XSEDE v8.2.12 and the GTR + I + G optimal model (Stamatakis et al. 2008). Markov chain Monte Carlo (MCMC) simulations were then run with two sets of four chains for 10,000,000 generations at a sampling frequency of 100 and a burn-in of 25,000 trees (25%). Tree topologies were visualized using MEGA v.6.0 (Tamura et al. 2011).

## Data Availability

The datasets presented in this study can be found in online repositories. The names of the repositories and the accession numbers can be found at: https://www.ncbi.nlm.nih.gov/genbank/ (OP292224, OP292225, OP292226, and OP292227).
